# The association between frequent alcohol drinking and opioid consumption after abdominal surgery: A retrospective analysis

**DOI:** 10.1371/journal.pone.0171275

**Published:** 2017-03-16

**Authors:** Sheng-Chin Kao, Hsin-I Tsai, Chih-Wen Cheng, Ta-Wei Lin, Chien-Chuan Chen, Chia-Shiang Lin

**Affiliations:** 1 Department of Anesthesiology, Mackay Memorial Hospital, Taipei City, Taiwan; 2 School of Medicine, National Yang-Ming University, Taipei City, Taiwan; 3 Department of Anesthesiology, Chang Gung Memorial Hospital, Linkou, Taoyuan City, Taiwan; 4 Department of Anesthesiology, National Yang-Ming University Hospital, Yilan City, Yilan County, Taiwan; Nanjing University Medical School Affiliated Nanjing Drum Tower Hospital, CHINA

## Abstract

**Aims:**

It is perceived that patients with a history of frequent alcohol consumption require more opioids for postoperative pain control and experience less postoperative nausea and vomiting than patients without such a history. However, there is scarce evidence supporting this notion. The aim of this study was to assess association between frequent alcohol consumption and opioid requirement for postoperative pain control and occurrence of postoperative nausea and vomiting.

**Methods:**

The medical records for 4143 patients using intravenous patient-control analgesia with opioids after abdominal surgery between January 2010 and September 2013 were obtained, and associations were sought between the cumulative opioid consumption (in intravenous morphine equivalence) per body weight (mg/kg) in the first 2 days after abdominal operation and several demographic and clinical variables by multiple regression analysis. The association between the occurrence of postoperative nausea and vomiting and several demographic and clinical variables was also sought by multiple logistic regression analysis.

**Results:**

Frequent alcohol drinking, among other previously reported factors, was associated with increased opioid consumption for postoperative pain control (*p* < 0.001). The estimate effect of frequent alcohol drinking was 0.117 mg/kg. Frequent alcohol drinking was also associated with decreased risks of postoperative nausea (odds ratio = 0.59, *p* = 0.003) and vomiting (odds ratio = 0.49, *p* = 0.026).

**Conclusions:**

Frequent alcohol drinking was associated with increased opioid consumption for postoperative pain control and decreased risks of postoperative nausea and vomiting after abdominal surgery.

## Introduction

Postoperative pain management is a clinical challenge. Inadequate pain relief after operation is associated with decreased patient satisfaction [1], delayed hospital discharge [2], adverse functional outcome [3], and increased morbidity and mortality [4]. Despite the advancement in our understanding of pathophysiology of nociception and development of novel analgesic techniques, opioids are still among the most commonly prescribed medications to treat moderate to severe postoperative pain. Although opioids are effective in relieving postoperative pain, there are great variations in opioid requirement to achieve adequate pain control among individual patient [5–9], and postoperative opioids is an major predictor of postoperative nausea and/or vomiting (PONV) [10–14]. Although PONV rarely leads to serious complications, it may decrease patient satisfaction and increase healthcare cost [11, 14]. Hence, knowledge regarding predicting factors of postoperative opioid requirement and of occurrence of opioid-related side effects such as PONV could be helpful in formulating an effective pain control program with minimized side effects for individual patient. Previous studies have examined numerous potential factors and identified age, types of surgery, pre-existing psychological distress, ethnicity, and genetic polymorphisms as predictors of opioid requirement after operation [5–9]. However, the literatures are insufficient in evaluating the impact of alcohol consumption on opioid consumption for pain control after a major operation. Similarly, early studies have examined numerous potential factors and identified age, gender, current smoking status, history of motion sickness or PONV, using volatile anesthetics and postoperative opioids as predictors of PONV [11–14], but there is only limited evaluation on the impact of alcohol consumption on the occurrence of PONV [10].

In fact, alcohol is one of the most commonly used substances around the world and is capable of exerting a multitude of impacts on pain perception and analgesic requirement. For example, chronic alcohol consumption can induce neuropathic pain through both central and peripheral mechanisms [15, 16]. In preclinical studies, acute administration of alcohol in animals can produce a moderate antinociceptive effect through interaction with the opioid receptor system in the central nervous system [17–19]. On the contrary, chronic alcohol consumption produced mechanical hyperalgesia and tolerance to opioid-induced antinociception in animal experiments [20, 21]. Clinically, it is perceived that patients with a history of frequent alcohol consumption require more opioids for postoperative pain control and experience less opioid-related side effects, such as nausea and vomiting than those without such a history. However, the clinical impact of chronic alcohol consumption on postoperative opioid consumption and occurrence of opioid-related side effects has been rarely addressed in previous studies [10]. The aim of this study was to evaluate whether frequent alcohol intake, in addition to other factors, is associated with an increased opioid requirement for postoperative pain control. Any association was also sought between frequent alcohol drinking and postoperative nausea and vomiting (PONV), because the occurrence of PONV has been attributed to postoperative administration of opioids [11–14].

## Materials and methods

After approval by the institutional review board (IRB) of Chang Gung Memorial hospital (approval number: 102-3855B), a retrospective analysis of patients using intravenous patient-controlled analgesia (ivPCA) for postoperative pain control in a single tertiary center between January 2010 and September 2013 was conducted. Informed consent was not required by the IRB, because the data were analyzed anonymously. As literature has demonstrated a positive correlation between abdominal surgery and postoperative analgesic consumption [6], only patients receiving abdominal surgery were included in the analysis. Those who received laparoscopic abdominal surgery, non-abdominal surgery, another operation within one week, partial agonists or antagonists of opioid receptors were excluded from the study. Patients who used opioid chronically or with incomplete data were also excluded from the study.

As the routine practice in our department, major abdominal surgeries were carried out under general anesthesia without using dexmedetomine, clonidine, or ketamine. General anesthesia was induced with intravenous fentanyl (1-3mcg/mg) and propofol (1-3mg/mg), and endotracheal intubation was facilitated by intravenous cis-atracurium (0.2–0.3mg/kg) or rocuronium (0.6–0.9mg/kg). General anesthesia was maintained by sevoflurane or desflurane inhalation with intermittent bolus of cisatracurium and/or fentanyl at the discretion of the anesthesiologists. Patients receiving combined general anesthesia and regional anesthesia were excluded from analysis as well as those received regional analgesia for postoperative pain control.

Before providing ivPCA to each patient, it has been the standard practice of our acute pain service to interview the patient or his/her family. During the interview, the acute pain service team inquires and records the following histories in a standardized form regarding medical illness (including presence or absence of cardiovascular, diabetic, pulmonary, renal, liver, and gastrointestinal diseases), recreational drug usage, history of drug allergy, current cigarette or tobacco smoking status, average frequency, type (such as wine, beer, and whisky) and amount of alcohol drinking, and chronic use of analgesics or psychotropic medications. The patient’s age, gender, and body weight as well as the site, type and approach (open or laparoscopy) of surgery the patient had received were also recorded in a standardized form together with the abovementioned histories. Intravenous patient-controlled analgesia (ivPCA) comprising of fentanyl or a mixture of fentanyl and morphine was provided for postoperative pain control after abdominal surgery when epidural analgesia was not adopted. Patients were instructed to use ivPCA if the intensity of pain was equal to or more than moderate on a verbal severity scale (no pain, mild pain, moderate pain, severe pain and extreme pain). Also as part of our standard practice, daily recording of cumulative opioid consumption from ivPCA was done starting postoperative day 1 in addition to the presence of opioid-related side effects including nausea, vomiting, dizziness, drowsiness, pruritus, and respiratory depression if any. If additional opioid other than from ivPCA was administered, the dosage was also recorded. These recorded data were saved as the acute pain service database.

Given multiple factors are associated with postoperative analgesic consumption [6–9] and occurrence of PONV [10–14], variables other than alcohol consumption were also included in the analysis. Candidate variables chosen for analysis included both demographic and clinical factors. Demographic factors included patients’ age, gender, histories of smoking and frequent alcohol consumption. Frequent alcohol consumption is defined as having 4 or more drinks per week or drinking alcohol on 4 or more days per week. A drink is defined as 12 oz of beer, 5 oz of wine, or 1.5 oz of liquor (approximately 14g alcohol). Clinical factors included American Society of Anesthesiologist physical status, surgical type, medical histories, and drug histories regarding drug allergy and chronic use of nonsteroid anti-inflammatory drugs (NSAIDs) or psychotrophic drugs. Medical histories were categorized into pulmonary disease, diabetes mellitus (DM), cardiovascular disease, gastrointestinal diseases, liver diseases, renal diseases, and end-stage renal disease under hemodialysis. Surgical procedures were grouped into colorectal, hepatobiliary, stomach, pancreatic, urological, and splenic surgery. Surgeries scheduled as exploratory laparotomy were excluded due to great variations.

Total opioid consumption per body weight (kg) during the first two days after operation was used as the outcome measurement to evaluate the potential association between opioid consumption and candidate variables. Total opioid consumption was calculated by adding opioids administered other than from ivPCA to cumulative opioids usage from ivPCA recorded on postoperative day 2. The amount of total opioids consumed was converted to intravenous morphine equivalence using the equianalgesic conversion ratios of meperidine: morphine = 75: 10 and fentanyl: morphine = 0.1: 10 [22, 23].

### Statistical analysis

Categorical data were expressed in number and percentage, and continuous data were expressed in mean and standard deviation. In analyzing association between total opioid consumption and potential factors, candidate categorical factors were initially examined by two sample independent *t*-test, or analysis of variance (ANOVA). Non-parametric tests were used in case of small size in any of the categories. Candidate factors found to have some evidence (*p*< 0.1) in the initial analyses were subsequently included in a multiple linear regression analysis.

In analyzing association between occurrence of PONV and potential factors, candidate categorical factors and continuous factors were initially examined by chi-square test and logistic regression analyses, respectively. Candidate factors found to have a *p*-value< 0.1in the initial analyses were included in subsequent multiple logistic regression analysis. A *p*-value of < 0.05 was considered statistically significant. All statistical analyses were performed using SPSS v17.

## Results

A total of 14,678 patients having used ivPCA for postoperative pain control between January 2010 and September 2013 were identified from the acute pain service database. Among them, 4143 patients were included in the analyses after exclusion criteria were applied and their demographic and clinical data were illustrated in Table 1.

**Table 1:** Demographic and clinical data of patients and univariate analysis of candidate factors potentially associated with opioid consumption after abdominal surgery.

	N (%)	Opioid consumption, mg/kg (mean ± SD)	*p*-value
Total	4143(100)	1.15 ± 0.43	
Body weight, kg (mean ± SD)	63.15 ±12.88		
Age, years (mean ± SD)	60.64 ±14.39		< 0.001
< 30	113 (2.7)	1.34 ± 0.47	
30–45	471 (11.4)	1.33 ± 0.53	
45–60	1343 (32.4)	1.18 ± 0.43	
60–75	1532 (37.0)	1.10 ± 0.39	
> 75	684 (16.5)	1.04 ± 0.39	
Gender			< 0.001
Female	2418 (58.4)	1.18 ± 0.44	
Male	1725 (41.6)	1.11 ± 0.42	
ASA			0.792
1	76 (1.8)	1.29 ± 0.42	
2	2589 (62.5)	1.18 ± 0.44	
3	1478 (35.7)	1.10 ± 0.41	
Alcohol drinking			< 0.001
Infrequent	3227 (77.9)	1.11 ± 0.40	
Frequent^a^	916 (22.1)	1.28 ± 0.50	
Current smoker			< 0.001
No	3006 (72.6)	1.11 ± 0.41	
Yes	1137 (27.4)	1.26 ± 0.47	
Surgical types			< 0.001
Hepatobiliary	1203 (29.0)	1.14 ± 0.41	
Colorectal	1673 (40.4)	1.13 ± 0.41	
Stomach	513 (12.4)	1.24 ± 0.46	
Pancreatic	210 (5.1)	1.32 ± 0.52	
Urological	504 (12.2)	1.11 ± 0.46	
Splenic	40 (1.0)	1.23 ± 0.42	
Medical history			
Pulmonary disease			0.367
No	3929 (94.8)	1.15 ± 0.43	
Yes	214 (5.2)	1.18 ± 0.50	
Cardiovascular disease			<0.001
No	2343 (56.6)	1.19 ± 0.45	
Yes	1800 (43.4)	1.09 ± 0.40	
Diabetes mellitus			<0.001
No	3365 (81.2)	1.16 ± 0.44	
Yes	778 (18.8)	1.10 ± 0.39	
Gastrointestinal disease			0.053
No	3142 (75.8)	1.14 ± 0.43	
Yes	1001 (24.2)	1.17 ± 0.44	
Hepatic disease			0.012
No	3085 (74.5)	1.16 ± 0.44	
Yes	1058 (25.5)	1.12 ± 0.41	
Renal disease			0.572
No	3785 (91.4)	1.15 ± 0.43	
Yes	358 (8.6)	1.14 ± 0.42	
ESRD under hemodialysis			0.859
No	4062 (98.0)	1.15 ± 0.43	
Yes	81 (2.0)	1.16 ± 0.43	
Drug history			
Chronic NSAIDs user			0.032
No	4085 (98.6)	1.15 ± 0.43	
Yes	58 (1.4)	1.30 ± 0.54	
Chronic Psychotrophic medication			0.027
No	3667 (88.5)	1.14 ± 0.42	
Yes	476 (11.5)	1.20 ± 0.51	
History of drug allergy			0.305
No	3658 (88.3)	1.15 ± 0.43	
Yes	485 (11.7)	1.14 ± 0.46	

ASA: American Society of Anesthesiologist physical status

ESRD: end stage renal disease

NSAIDs: non-steroid anti-inflammatory drugs.

^a^Frequent alcohol drinking is defined as having 4 or more drinks per week or drinking alcohol on 4 or more days per week. A drink is defined as 12 oz of beer, 5 oz of wine, or 1.5 oz of liquor (approximately 14g alcohol)

### Factors associated with increased postoperative opioid consumption

Univariate analyses revealed that male gender, frequent alcohol drinking, current smoker, certain surgical types, chronic use of NSAIDs, chronic use of psychotrophic medications, and gastrointestinal diseases were associated with increased opioid consumption. On the other hand, cardiovascular diseases, diabetes mellitus and hepatic diseases were associated with decreased opioid consumption (Table 1). Age was inversely related to the total opioid consumption. Subsequent multiple regression analysis revealed that young age, frequent alcohol drinking, positive smoking status, stomach or pancreatic surgery and chronic use of NSAIDs or psychotrophic medications were associated with increased opioid consumption, while cardiovascular and hepatic disease were associated with decreased opioid consumptions (Table 2).

**Table 2:** Multiple regression analysis of opioid consumption after abdominal surgery.

	Estimate (mg/kg)	95% CI	*p*-value
Age	-0.005	-0.006 to -0.005	<0.001
Female	-0.006	-0.036 to 0.023	0.669
Frequent alcohol drinking^a^	0.117	0.082 to 0.151	<0.001
Current smoker	0.082	0.048 to 0.115	<0.001
Surgical types			
Hepatobiliary	0		
Colorectal	0.008	-0.026 to 0.042	0.641
Stomach	0.108	0.062 to 0.153	<0.001
Pancreatic	0.187	0.125 to 0.248	<0.001
Urological	-0.024	-0.069 to 0.021	0.288
Splenic	0.100	-0.029 to 0.230	0.129
Cardiovascular disease	-0.031	-0.059 to -0.003	0.031
Diabetes Mellitus	-0.020	-0.053 to 0.014	0.252
Gastrointestinal disease	0.029	-0.001 to 0.058	0.060
Hepatic disease	-0.043	-0.076 to -0.011	0.009
Chronic use of NSAIDs	0.112	0.005 to 0.219	0.040
Chronic use of psychotrophic medications	0.087	0.047 to 0.127	<0.001

NSAIDs: non-steroid anti-inflammatory drugs.

^a^Frequent alcohol drinking is defined as having 4 or more drinks per week or drinking alcohol on 4 or more days per week. A drink is defined as 12 oz of beer, 5 oz of wine, or 1.5 oz of liquor (approximately 14g alcohol)

### Factors associated with postoperative nausea

Univariate analysis revealed that young age, female gender, urological surgery, and a history of drug allergy were associated with increased risks of postoperative nausea, while frequent alcohol drinking, current smoker, stomach surgery, cardiovascular and hepatic disease were associated with decreased risks of postoperative nausea (Table 3). Subsequent multiple logistic regression analysis revealed that female gender was associated with increased risks of postoperative nausea while frequent alcohol drinking and stomach surgery were associated with decreased risks of postoperative nausea (Table 4).

**Table 3:** Univariate analysis of candidate factors potentially associated with occurrence of nausea after abdominal surgery.

	Odds ratio	95% CI	*p*-value
Age, years	0.99	0.99 to 1.00	0.026
Gender			
Male	1		
Female	2.70	2.23 to 3.27	<0.001
Alcohol drinking			
Frequent^a^	0.39	0.29 to 0.52	<0.001
Infrequent	1		
Current smoker			
Yes	0.51	0.40 to 0.65	<0.001
No	1		
Surgical types			
Hepatobiliary	1		
Colorectal	1.14	0.91 to 1.43	0.253
Stomach	0.60	0.41 to 0.88	0.008
Pancreatic	0.79	0.48 to 1.30	0.358
Urological	1.55	1.16 to 2.08	0.003
Splenic	1.08	0.42 to 2.79	0.879
Medical history			
Pulmonary disease			
Yes	0.81	0.51 to 1.27	0.356
No	1		
Cardiovascular disease			
Yes	0.80	0.66 to 0.96	0.019
No	1		
Diabetes mellitus			
Yes	0.86	0.67 to 1.10	0.239
No	1		
Gastrointestinal disease			
Yes	0.85	0.68 to 1.06	0.146
No	1		
Hepatic disease			
Yes	0.76	0.61 to 0.95	0.016
No	1		
Renal disease			
Yes	1.00	0.72 to 1.39	0.994
No	1		
ESRD under hemodialysis			
Yes	0.78	0.37 to 1.62	0.503
No	1		
Drug history			
Chronic NSAIDs user			
Yes	0.39	0.12 to 1.24	0.109
No	1		
Chronic Psychotrophic medication			
Yes	0.94	0.70 to 1.27	0.690
No	1		
History of drug allergy			
Yes	1.54	1.19 to 1.99	0.001
Never	1		

ASA: American Society of Anesthesiologist physical status

ESRD: end stage renal disease

NSAIDs: non-steroid anti-inflammatory drugs.

^a^Frequent alcohol drinking is defined as having 4 or more drinks per week or drinking alcohol on 4 or more days per week. A drink is defined as 12 oz of beer, 5 oz of wine, or 1.5 oz of liquor (approximately 14g alcohol)

**Table 4:** Multiple logistic regression analysis of occurrence of nausea after abdominal surgery.

	Odds ratio	95% CI	*p*-value
Age	1.00	0.99 to 1.00	0.245
Female	2.19	1.75 to 2.74	<0.001
Frequent alcohol drinking^a^	0.59	1.18 to 2.28	0.003
Current smoker	0.93	0.70 to 1.25	0.644
No Cardiovascular disease	1.02	0.74 to 1.41	0.916
No Hepatic disease	1.23	0.89 to 1.70	0.212
History of drug allergy	2.56	0.79 to 8.30	0.118
Surgical types			
Hepatobiliary	1		
Colorectal	0.99	0.77 to 1.28	0.962
Stomach	0.56	0.38 to 0.83	0.004
Pancreatic	0.66	0.40 to 1.10	0.110
Urological	1.31	0.96 to 1.79	0.092
Splenic	0.74	0.28 to 1.95	0.543

^a^Frequent alcohol drinking is defined as having 4 or more drinks per week or drinking alcohol on 4 or more days per week. A drink is defined as 12 oz of beer, 5 oz of wine, or 1.5 oz of liquor (approximately 14g alcohol).

### Factors associated with postoperative vomiting

Univariate analysis revealed that female gender and urological surgery were associated with increased risks of postoperative vomiting, while frequent alcohol drinking, current smoker and stomach surgery were associated with decreased risks of postoperative vomiting (Table 5). Subsequent multiple logistic regression analysis revealed that female gender and urological surgery were associated with increased risks of postoperative vomiting, while frequent alcohol drinking and stomach surgery were associated with decreased risks of postoperative vomiting (Table 6).

**Table 5:** Univariate analysis of candidate factors potentially associated with occurrence of vomiting after abdominal surgery.

	Odds ratio	95% CI	*p*-value
Age, years	0.99	0.98 to 1.00	0.113
Sex			
Male	1		
Female	3.88	2.79 to 5.40	<0.001
Alcohol drinking			
Frequent^a^	0.26	0.15 to 0.45	<0.001
Infrequent	1		
Current smoker			
Yes	0.42	0.28 to 0.64	<0.001
No	1		
Surgical types			
Hepatobiliary	1		
Colorectal	1.07	0.73 to 1.55	0.735
Stomach	0.48	0.24 to 0.95	0.036
Pancreatic	0.71	0.30 to 1.68	0.433
Urological	2.48	1.63 to 3.76	<0.001
Splenic	1.27	0.30 to 5.41	0.749
Medical history			
Pulmonary disease			
Yes	1.18	0.63 to 2.20	0.609
No	1		
Cardiovascular disease			
Yes	0.87	0.64 to 1.18	0.366
No	1		
Diabetes mellitus			
Yes	0.91	0.61 to 1.34	0.624
No	1		
Gastrointestinal disease			
Yes	0.76	0.52 to 1.09	0.138
No	1		
Hepatic disease			
Yes	0.72	0.50 to 1.05	0.086
No	1		
Renal disease			
Yes	1.08	0.65 to 1.80	0.766
No	1		
ESRD under hemodialysis			
Yes	1.75	0.75 to 4.07	0.196
No	1		
Drug history			
Chronic NSAIDs user			
Yes	1.18	0.36 to 3.80	0.785
No	1		
Chronic Psychotrophic medication			
Yes	1.05	0.66 to 1.65	0.845
No	1		
History of drug allergy			
Yes	1.14	0.73 to 1.77	0.562
Never	1		

ASA: American Society of Anesthesiologist physical status

ESRD: end stage renal disease

NSAIDs: non-steroid anti-inflammatory drugs.

^a^Frequent alcohol drinking is defined as having 4 or more drinks per week or drinking alcohol on 4 or more days per week. A drink is defined as 12 oz of beer, 5 oz of wine, or 1.5 oz of liquor (approximately 14g alcohol).

**Table 6:** Multiple logistic regression analysis of occurrence of vomiting after abdominal surgery.

	Odds ratio	95% CI	*p*-value
Female	3.13	2.14 to 4.58	<0.001
Frequent alcohol drinking^a^	0.49	1.09 to 3.84	0.026
Current smoker	1.01	0.62 to 1.66	0.961
Surgical types			
Hepatobiliary	1		
Colorectal	0.93	0.64 to 1.36	0.701
Stomach	0.46	0.23 to 0.92	0.028
Pancreatic	0.59	0.25 to 1.40	0.231
Urological	2.05	1.34 to 3.14	<0.001
Splenic	0.90	0.21 to 3.86	0.883

^a^Frequent alcohol drinking is defined as having 4 or more drinks per week or drinking alcohol on 4 or more days per week. A drink is defined as 12 oz of beer

5 oz of wine, or 1.5 oz of liquor (approximately 14g alcohol).

## Discussion

Similar to previous reports, our current analyses have revealed that young age, smoking, and upper abdominal surgeries (stomach and pancreatic surgeries) as significant predictors of increased opioid consumption for postoperative pain control [6–9]. In addition, our current analyses further identified frequent alcohol drinking as a significant predictor of postoperative opioid consumption. The exact mechanisms underlying the association between frequent alcohol drinking and increased postoperative opioid consumption are unclear. In animal experiments, chronic alcohol consumption was associated with mu-opioid receptors dysfunction in the spinal cord [20] and produced tolerance to opioid antinociception through inhibition of mu-opioid receptor endocytosis [21]. Therefore, it is likely that the increased opioid consumption for postoperative pain control in frequent alcohol drinkers might result from alcohol-induced tolerance to opioid analgesic effect. The mechanisms underlying alcohol-induced tolerance to opioid analgesic effect remain unknown. In a recent review, robust evidence has been provided to demonstrate that chronic alcohol drinking activates the N-methyl-D-aspartate receptor (NMDAR) signaling and enhances synaptic plasticity in the brain, which greatly contributes to the alcohol-related behaviors [24]. Altered NMDAR activity has also been observed in the spinal cord after chronic alcohol exposure [25]. Given that enhanced activity of NMDAR also plays a pivotal role in the development of morphine tolerance induced by repeated morphine administration [26], it is highly likely that chronic alcohol consumption produced tolerance to opioid analgesic effects through activating NMDAR signaling in the central nervous system. Further research on the role of NMDAR signaling in the development of alcohol-induced tolerance to opioid analgesic effect is warranted.

In agreement with previous studies, our analyses have identified female gender [10–14] and urological surgery [12] as risk factors of PONV. In addition, our analyses suggested that frequent alcohol drinking may be protective against PONV. Similar result on postoperative nausea was reported previously [20]. The mechanisms underlying PONV were not clearly understood. Based on clinical studies, the use of volatile anesthetics may be the main cause of PONV in the early (2–6 hours) postoperative period, while postoperative opioids may account for PONV in the delayed postoperative period [13, 14]. Since only PONV occurred in POD1 and POD2 (delayed postoperative period) were recorded and analyzed in this current study, it is likely that frequent alcohol drinking could exert a protective effect against opioid-induced nausea and vomiting. Contrary to previous reports [10–11], current smoking status is not associated with a lower risk of PONV in our analyses. Similar result has been reported in recent studies [27, 28]. The reason of this discrepancy is unknown.

Our study also revealed other predicting factors of postoperative opioid consumption that were not previously reported. Among these factors, pancreatic surgery was associated with the most prominent effect on postoperative opioid consumption. This may be related to the large surgical wound and profound tissue injury associated with pancreatic surgery. Although the association between cardiovascular and hepatic diseases with postoperative opioid consumption was statistically significant, their clinical significance seemed negligible when considering their small estimate effects (0.03 and0.04 mg/kg morphine in 2 days).

In current analyses, the occurrence of PONV was less frequent after stomach surgery. Mu opioid receptors have been identified on nerve terminals in myenteric plexus of the stomach [29]. It is possible that the removal of these peripheral opioid receptors during gastrectomy contributed to the decreased occurrence of PONV after stomach surgery. Further study is required to investigate this hypothesis.

Our analyses have several limitations. First, although collecting information regarding the frequency and amount of alcohol consumption was mandatory before instituting the ivPCA for each patient, we did not specifically collect information regarding the duration of such drinking behavior as well as blood level of alcohol. Therefore, the cumulating alcohol exposure could not be estimated. In addition, we categorized alcohol consumption into dichotomous variables as frequent and infrequent alcohol drinking, precluding a precise measurement of alcohol consumption (e.g., amount, frequency) in relation to opioid consumption for postoperative pain control. Hence, we could not quantitatively determine the minimal duration, frequency, or amount of alcohol consumption required to produce tolerance to opioid analgesic effects or opioid-related PONV in humans. In rats, tolerance to opioid antinociception developed after 8 weeks, but not 4 weeks, of alcohol consumption [21] and persisted up to 24 days after alcohol withdrawal [20]. Second, psychological distress was reported as a significant predictor of postoperative analgesic consumption [6], but preoperative psychological evaluation was not available in our analyses. However, a history of chronic use of psychotropic medication might serve as a surrogate indicator of psychological distress. Indeed, in line with previous report, our analyses showed positive association between chronic use of psychotropic medications and increased opioid consumption. Third, pharmacogenetic analyses were not performed; the genetic factors might impact on postoperative opioid requirement [7, 8]. Fourth, histories regarding motion sickness and PONV, two well-recognized risk factors of PONV, were not available in our reviewed medical records and therefore were not included in our analyses. Fifth, we did not include intraoperative opioid doses as a potential factor in the analysis, which, in high doses, have been associated with increased postoperative morphine consumption [30]. However, the impact of intraoperative opioids on postoperative opioid consumption is still controversial [31] and if exists, seems to be limited to remifentanil and cannot to be extrapolated to other opioids such as fentanyl and sufentanil [30]. Since only fentanyl was used intraoperatively and remifentanil was not available in our institution, we assume that not including intraoperative opioids in our current analysis might not have significant impact on the result regarding postoperative opioid consumption. Similarly, in a recent meta-analysis, intraoperative opioid is not an independent predictor of PONV [11]. Therefore, not including intraoperative opioids in our analysis might not impact the result regarding occurrence of PONV. Finally, the duration of operation, invasiveness of specific operative methods, degree of postoperative alertness, postoperative admission to ward or intensive care unit (ICU), length of ICU stay, duration of mechanical ventilation, and timing of extubation may also impact the postoperative opioid consumption and PONV but were not included in the analysis.

In conclusion, frequent alcohol drinkers consumed an increased amount of opioids for postoperative pain control but were less likely to suffer from PONV. Frequent alcohol drinking possibly produced tolerance to both opioid analgesic effect and opioid-related nausea and vomiting. Further prospective studies are required to exam this hypothesis.

## References

[1] Gerbershagen HJ, Aduckathil S, van Wijck AJ, Peelen LM, KalkmanCJ, Meissner W. Pain intensity on the first day after surgery: a prospective cohort study comparing 179 surgical procedures. Anesthesiology. 2013;118:934–944 10.1097/ALN.0b013e31828866b3 23392233

[2] Wang H, Boctor B, Verner J. The effect of single-injection femoral nerve block on rehabilitation and length of hospital stay after total knee replacement. Reg Anesth Pain Med. 2002;27:139–144. 11915059 10.1053/rapm.2002.29253

[3] Ryu J, Saito S, Yamamoto K, Sano S. Factors influencing the postoperative range of motion in total knee arthroplasty. Bull Hosp Jt Dis. 1993;53:35–40. 8012266

[4] Rodgers A, Walker N, Schug S, McKee A, Kehlet H, van Zundert A, et al: Reduction of postoperative mortality and morbidity with epidural or spinal anaesthesia: Results from overview of randomized trials. BMJ 2000; 321:1493 11118174 10.1136/bmj.321.7275.1493 PMC27550

[5] Wu CL, Raja SN. Treatment of acute postoperative pain. Lancet. 2011; 377:2215–2225. 10.1016/S0140-6736(11)60245-6 21704871

[6] Ip HY, Abrishami A, Peng PW, Wong J, Chung F. Predictors of postoperative pain and analgesic consumption: a qualitative systematic review. Anesthesiology. 2009;111: 657–677 10.1097/ALN.0b013e3181aae87a 19672167

[7] Yoshida K, Nishizawa D, Ichinomiya T, Ichinohe T, Hayashida M, Fukuda K, et al Prediction formulas for individual opioid analgesic requirements based on genetic polymorphism analyses. PLoS One. 2015 1 23;10(1):e0116885 10.1371/journal.pone.0116885 25615449 PMC4304713

[8] Hwang IC, Park JY, Myung SK, Ahn HY, Fukuda K, Liao Q. OPRM1 A118G gene variant and postoperative opioid requirement: a systematic review and meta-analysis. Anesthesiology. 2014;121:825–834. 10.1097/ALN.0000000000000405 25102313

[9] Weingarten TN, Sprung J, Flores A, Baena AM, Schroeder DR, Warner DO. Opioid requirements after laparoscopic bariatric surgery. Obes Surg. 2011;21:1407–1412. 10.1007/s11695-010-0217-9 20563662

[10] Morino R, Ozaki M, Nagata O, Yokota M. Incidence of and risk factors for postoperative nausea and vomiting at a Japanese Cancer Center: first large-scale study in Japan. J Anesth. 2013;27:18–24. 10.1007/s00540-012-1468-5 22923285 PMC3574566

[11] Apfel CC, Heidrich FM, Jukar-Rao S, Jalota L, Hornuss C, Whelan RP, et al Evidence-based analysis of risk factors for postoperative nausea and vomiting. Br J Anaesth. 2012;109:742–753. 10.1093/bja/aes276 23035051

[12] Stadler M, Bardiau F, Seidel L, Albert A, Boogaerts JG. Difference in risk factors for postoperative nausea and vomiting. Anesthesiology. 2003;98:46–52. 12502978 10.1097/00000542-200301000-00011

[13] Apfel CC, Kranke P, Katz MH, Goepfert C, Papenfuss T, Rauch S, et al Volatile anaesthetics may be the main cause of early but not delayed postoperative vomiting: a randomized controlled trial of factorial design. Br J Anaesth. 2002;88:659–668. 12067003 10.1093/bja/88.5.659

[14] Gan TJ. Risk factors for postoperative nausea and vomiting. Anesth Analg. 2006;102:1884–98. 10.1213/01.ANE.0000219597.16143.4D 16717343

[15] Miyoshi K1, Narita M, Takatsu M, Suzuki T. mGlu5 receptor and protein kinase C implicated in the development and induction of neuropathic pain following chronic ethanol consumption. Eur J Pharmacol. 2007; 562: 208–11. 10.1016/j.ejphar.2007.01.091 17349994

[16] Koike H, Sobue G. Alcoholic neuropathy. Curr Opin Neurol. 2006;19: 481–6. 10.1097/01.wco.0000245371.89941.eb 16969158

[17] Campbell VC1, Taylor RE, Tizabi Y. Effects of selective opioid receptor antagonists on alcohol-induced and nicotine-induced antinociception. Alcohol Clin Exp Res. 2007 8;31:1435–40. 10.1111/j.1530-0277.2007.00432.x 17550364

[18] Mogil JS, Marek P, Yirmiya R, et al Antagonism of the non-opioid component of ethanol-induced analgesia by the NMDA receptor antagonist MK-801. Brain Res 1993; 602: 126–30. 8448649 10.1016/0006-8993(93)90251-h

[19] Gatch MB. Ethanol withdrawal and hyperalgesia. Curr Drug Abuse Rev. 2009; 2: 41–50. 19630736 10.2174/1874473710902010041

[20] Narita M, Miyoshi K, Narita M, Suzuki T. Functional reduction in mu-opioidergic system in the spinal cord under a neuropathic pain-like state following chronic ethanol consumption in the rat. Neuroscience. 2007;144:777–782. 10.1016/j.neuroscience.2006.10.028 17156932

[21] He L, Whistler JL (2011) Chronic ethanol consumption in rats produces opioid antinociceptive tolerance through inhibition of mu opioid receptor endocytosis. PLoS One. 2011;6(5):e19372 10.1371/journal.pone.0019372 21602922 PMC3094338

[22] Chung KC, Barlev A, Braun AH, Qian Y, Zagari M. Assessing analgesic use in patients with advanced cancer: development of a new scale—the Analgesic Quantification Algorithm. PainMed. 2014;15:225–232. 10.1111/pme.12299 24400921

[23] Shaheen PE, Walsh D, Lasheen W, Davis MP, Lagman RL. Opioid equianalgesic tables: are they all equally dangerous? J Pain Symptom Manage. 2009; 38:409–417. 10.1016/j.jpainsymman.2009.06.004 19735901

[24] Morisot N, Ron D. Alcohol-Dependent Molecular Adaptations of the NMDA Receptor System. Genes Brain Behav. 2016 12 1. 10.1111/gbb.12363 PMC5444330 27906494

[25] Narita M1, Miyoshi K, Narita M, Suzuki T. Changes in function of NMDA receptor NR2B subunit in spinal cord of rats with neuropathy following chronic ethanol consumption. Life Sci. 2007; 80:852–9. 10.1016/j.lfs.2006.11.015 17156796

[26] Mayer DJ, Mao J, Holt J, Price DD. Cellular mechanisms of neuropathic pain, morphine tolerance, and their interactions. Proc Natl Acad Sci U S A. 1999; 96: 7731–6 10393889 10.1073/pnas.96.14.7731 PMC33610

[27] Brettner F, Janitza S, Prüll K, Weninger E, Mansmann U, Küchenhoff H, et al, Gender-Specific Differences in Low-Dose Haloperidol Response for Prevention of Postoperative Nausea and Vomiting: A Register-Based Cohort Study. PLoS One. 2016 1 11;11(1):e0146746 10.1371/journal.pone.0146746 26751066 PMC4713839

[28] Aydogan MS, Ozturk E, Erdogan MA, Yucel A, Durmus M, Ersoy MO, et al The effects of secondhand smoke on postoperative pain and fentanyl consumption. J Anesth. 2013;27:569–574. 10.1007/s00540-013-1565-0 23397133

[29] Smith HS, Laufer A. Opioid induced nausea and vomiting. Eur J Pharmacol. 2014; 722:67–78. 10.1016/j.ejphar.2013.09.074 24157979

[30] Fletcher D, Martinez V. Opioid-induced hyperalgesia in patients after surgery: a systematic review and a meta-analysis. Br J Anaesth. 2014; 112:991–1004. 10.1093/bja/aeu137 24829420

[31] Treskatsch S1, Klambeck M, Mousa SA, Kopf A, Schäfer M. Influence of high-dose intraoperative remifentanil with or without amantadine on postoperative pain intensity and morphine consumption in major abdominal surgery patients: a randomised trial. Eur J Anaesthesiol. 2014; 31:41–9. 10.1097/01.EJA.0000434967.03790.0e 24136378

